# Case report: Covered stent placement to treat delayed aneurysmal rupture after flow diverter-assisted coil embolization

**DOI:** 10.3389/fneur.2022.964733

**Published:** 2022-11-07

**Authors:** Xiheng Chen, Siming Gui, Linggen Dong, Longhui Zhang, Huijian Ge, Peng Liu, Youxiang Li, Ming Lv

**Affiliations:** ^1^Department of Interventional Neuroradiology, Beijing Neurosurgical Institute, Capital Medical University, Beijing, China; ^2^Department of Interventional Neuroradiology, Beijing Tian Tan Hospital, Capital Medical University, Beijing, China; ^3^Beijing Engineering Research Center for Interventional Neuroradiology, Beijing, China

**Keywords:** intracranial aneurysm, flow diverter, delayed aneurysm rupture, subarachnoid hemorrhage, case report

## Abstract

**Introduction:**

Flow diverter (FD) placement is widely accepted as a treatment for large saccular intracranial aneurysms. Delayed aneurysmal rupture (DAR) after FD placement is potentially catastrophic and difficult to treat. To our knowledge, using a Willis covered stent (WCS) to treat DAR after placement of a Pipeline Flex embolization device (PFED) combined with coiling has not been previously reported.

**Case presentation:**

A 49-year-old woman with an incidental asymptomatic large right supraclinoid internal carotid artery aneurysm was treated with PFED placement and adjunctive coiling. DAR causing subarachnoid hemorrhage occurred 11 hours after the procedure. Treatment using a WCS was successful and resulted in a favorable clinical outcome (modified Rankin scale score 2).

**Conclusion:**

DAR after FD implantation requires isolation of the aneurysm from the cerebral circulation as soon as possible. WCS placement can achieve this immediately and occlude the aneurysm. We hope our case could provide new idea for similar cases in the future.

## Introduction

Intracranial aneurysms are common and affect between 1 and 5% of the population, irrespective of ethnicity or geographical location (1). Flow diverter (FD) is an essential tool in endovascular treatment of large and giant intracranial aneurysms that can achieve satisfactory clinical and angiographic outcomes (2). However, it is associated with several complications, particularly delayed aneurysm rupture (DAR), which may cause severe neurological dysfunction or death (3). The mechanism of DAR after FD placement is not known. To our knowledge, no effective means of prevention has been established. Surgical and endovascular treatments of subarachnoid hemorrhage (SAH) and/or intracerebral hemorrhage (ICH) resulting from DAR are unsatisfactory and treatment guidelines are lacking.

In contrast to the immediate effect of surgical clipping or endovascular coiling, the treatment effect of FD placement involves parent vessel remodeling and occurs over time. In the interim, any residual filling within an aneurysm treated is theoretically associated with risk of rupture. Complete aneurysm thrombosis and early isolation from the cerebral circulation can prevent late rupture after FD placement (4). The Willis covered stent (WCS; MicroPort, Shanghai, China) was designed for parent vessel reconstruction and has been approved for treatment of intracranial aneurysms. This device can exclude aneurysms from the circulation and promote their immediate occlusion. Moreover, its efficacy and safety have been established for treatment of distal internal carotid artery (ICA) aneurysms, recurrent intracranial aneurysms after coiling, and large or giant aneurysms (5, 6).

We report a case of delayed rupture in a large supraclinoid ICA aneurysm that had been previously treated with placement of a Pipeline Flex embolization device (PFED; Medtronic, Dublin, Ireland) combined with adjunctive coiling. Treatment using a WCS during the acute rupture period successfully isolated the aneurysm from the cerebral circulation.

## Case presentation

**Figure 1:**
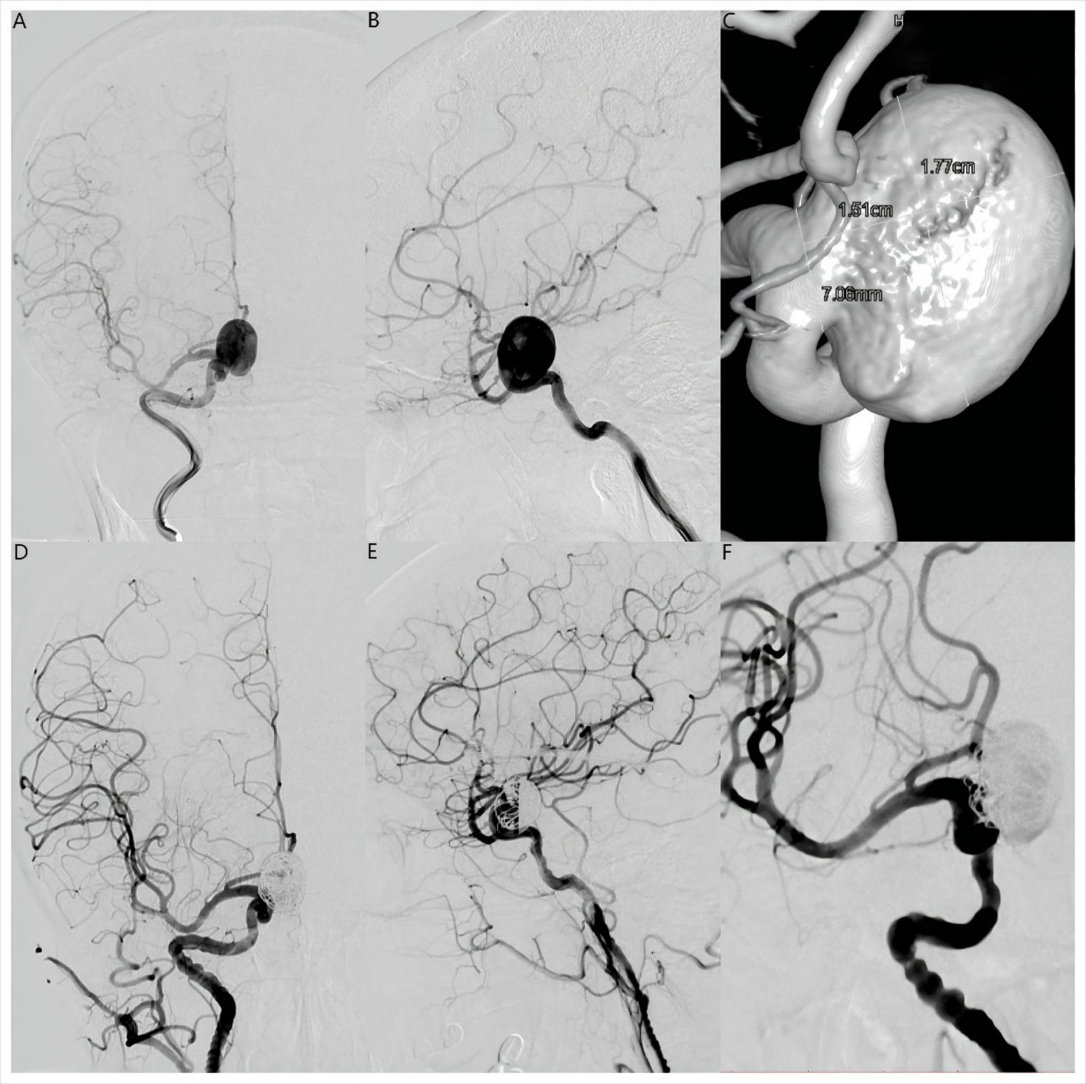
Anteroposterior **(A)** and lateral **(B)** digital subtraction angiography views and three-dimensional reconstruction angiography **(C)** revealed a large saccular right supraclinoid internal carotid artery aneurysm. Maximum diameter was 17.7 mm and the aneurysm neck was 7.06 mm. Anteroposterior **(D)**, lateral **(E)**, and operating position **(F)** angiographic views immediately after placement of a Pipeline Flex embolization device (Medtronic, Dublin, Ireland) showed contrast filling in the aneurysm lumen with entry remnant (O'Kelly-Marotta Grade C).

A 49-year-old woman was found to have an incidental asymptomatic large saccular right supraclinoid ICA aneurysm on computed tomography angiography (CTA) of the head at an outside hospital and was transferred to our center for further treatment. Cerebral angiography confirmed a large right supraclinoid ICA aneurysm (Figures 1A–C). Management options were discussed within our multidisciplinary team of neurosurgeons and interventional neuroradiologists. Flow diversion with adjunctive coiling was selected.

**Figure 2:**
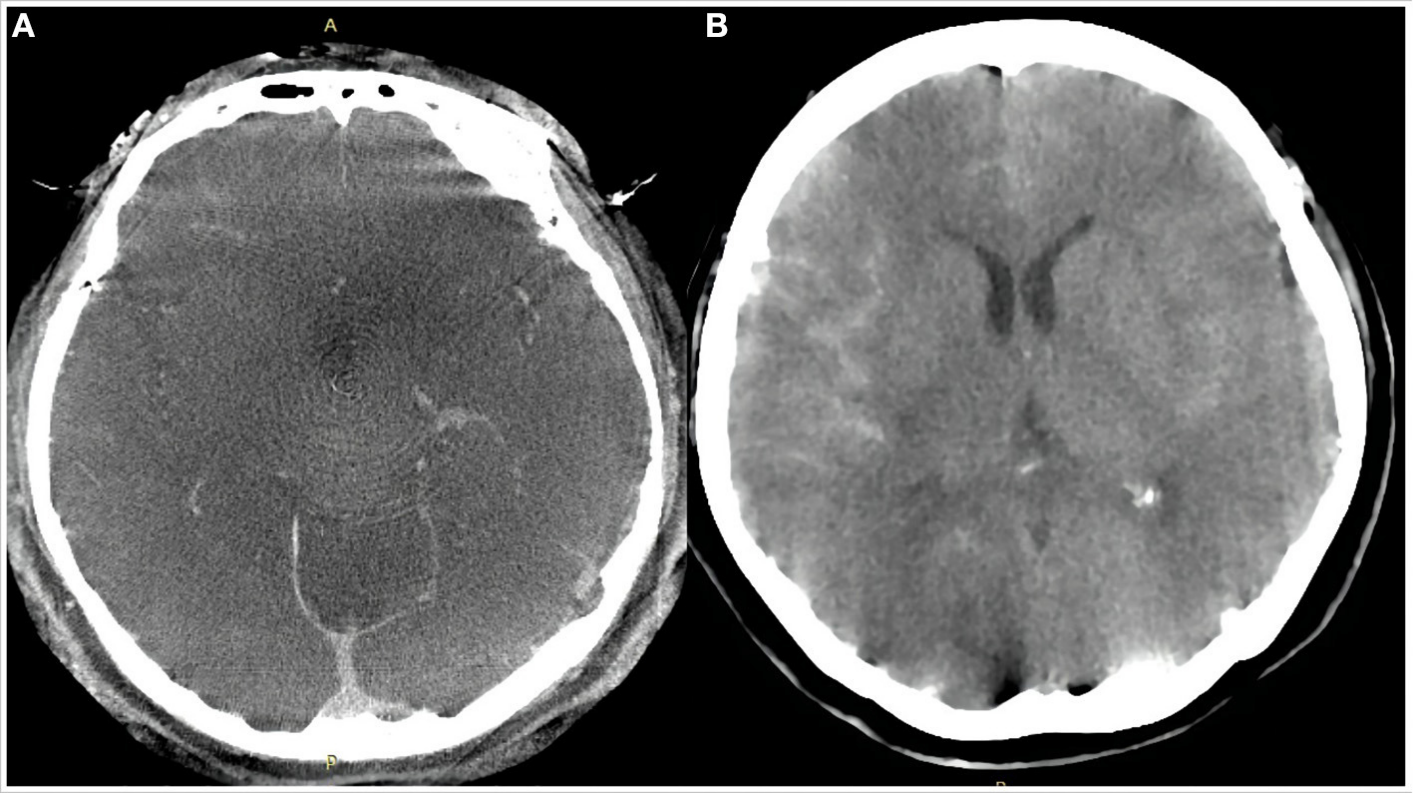
Dyna computed tomography immediately after the procedure **(A)** showed no subarachnoid hemorrhage or intracerebral hemorrhage. Emergency head computed tomography 11 h after the procedure **(B)** showed a small amount of hemorrhage in the Sylvian cistern, anterior interhemispheric cistern, and lateral ventricle.

Dual antiplatelet treatment (aspirin 100 mg daily and clopidogrel 75 mg daily) was initiated 5 days before the endovascular procedure. Platelet function was assessed by standard light transmittance aggregometry to measure platelet aggregation. Light transmittance aggregometry was conducted using platelet-rich plasma by the turbidimetric method in a 4-channel aggregometer (AG800; Techlink Biomedical, Inc, Beijing, China). Maximal platelet aggregation (MPA) was defined as the percentage change in light transmittance. The testing results showed that the MPA of ADP and arachidonic acid (AA) was 43.8 and 6.7%, respectively, suggesting that the platelet function of this patient was in a safe interval before therapeutic procedure. Under general anesthesia, a 6F 115 cm Navien (Covidien, Irvine, California, USA) intracranial support catheter assisted by a 6F 90 cm Neuron Max 088 sheath (Penumbra, Alameda, California, USA) was advanced into the right ICA. Under roadmap guidance, a 0.027-inch 150 cm Phenom 27 (Medtronic, MN, USA) microcatheter was navigated far into the middle cerebral artery (MCA) assisted by a 0.014-inch 200 cm Synchro-14 microwire (Stryker Neurovascular, Fremont, CA). A 0.017-inch 150 cm Echelon-10 (Medtronic, MN, USA) microcatheter was advanced into the aneurysmal sac for coiling. The PFED (4.5 mm × 25 mm) was then deployed from the M1 segment of the MCA to the C6 segment of the ICA. Two coils (25 × 50 mm, 22 × 50 mm, Axium Prime (Medtronic, Irvine, CA)) were deployed through the Echelon-10 microcatheter. Immediately after coiling, angiography showed near-complete occlusion with entry remnant (O'Kelly–Marotta grade C) (7) and Dyna-computed tomography (Dyna CT) showed no SAH or ICH (Figures 1D–F, 2A).

**Figure 3:**
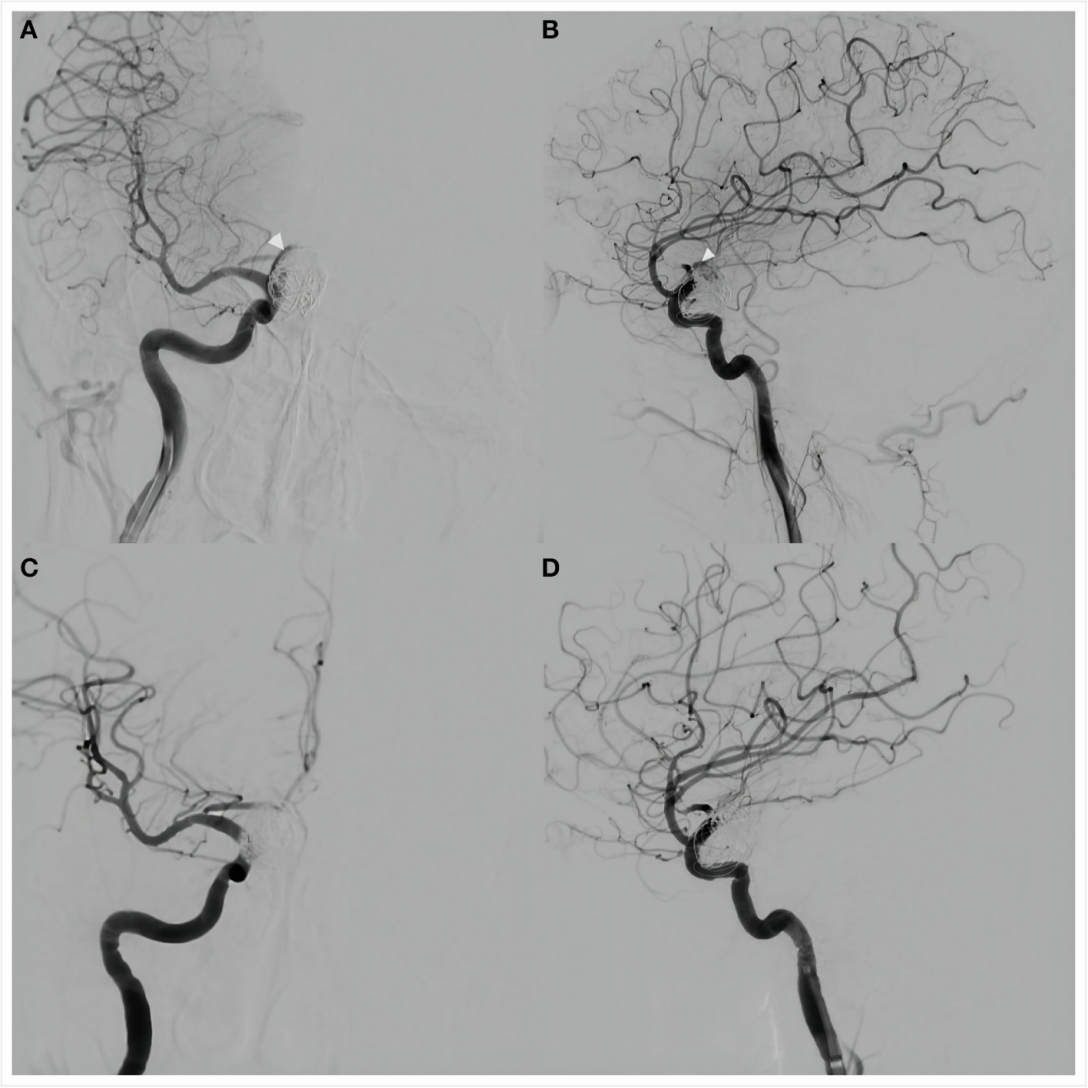
Anteroposterior **(A)** and lateral **(B)** views of cerebral angiography showed a small amount of blood flow into the aneurysm lumen (white arrows), suggesting delayed aneurysm rupture. Placement of a Willis covered stent (MicroPort, Shanghai, China) was then performed. Anteroposterior **(C)** and lateral **(D)** views of angiography performed immediately after the procedure showed no residual blood flow in the aneurysm lumen. The internal carotid and anterior choroidal arteries were patent.

Eleven hours after the procedure, the patient was found unresponsive. Emergency head CT showed SAH in the right Sylvian cistern, anterior interhemispheric fissure, and lateral ventricle (Hunt–Hess grade IV; modified Fisher grade 2; Figure 2B). Delayed aneurysm rupture was considered as the cause, and digital subtraction angiography showed that there was still a small amount of blood flow into the lumen of the aneurysm, suggesting the location of delayed aneurysm rupture here (Figures 3A,B). After a rapid discussion, the multidisciplinary team elected to proceed with endovascular placement of a WCS to completely isolate the aneurysm from the cerebral circulation.

### Therapeutic intervention

The procedure was performed under general anesthesia. A 5F 115 cm Navien intracranial support catheter assisted by a 6F 90 cm Neuron Max 088 sheath was passed into the right ICA through the right femoral artery and a 0.014-inch 205 cm Transcend EX (Boston Scientific/Target Therapeutics) microwire was advanced carefully through the PFED into the M2 segment of the right MCA through a 0.027-inch 150 cm Excelsior XT-27 (Stryker, Fremont, California, USA) microcatheter. After retrieval of the microcatheter, a 4.0 × 10 mm WCS was pushed slowly over the microwire and anchored in the aneurysmal neck without covering the ipsilateral anterior choroidal artery. Cerebral angiography was performed to confirm stent location before it was slowly inflated with 6 atm of pressure under fluoroscopic visualization. Angiography immediately after deflation of the balloon showed no aneurysmal filling and parent artery patency (Figures 3C,D). The patient was transferred to the intensive care unit after the procedure.

### Outcome

**Figure 4:**
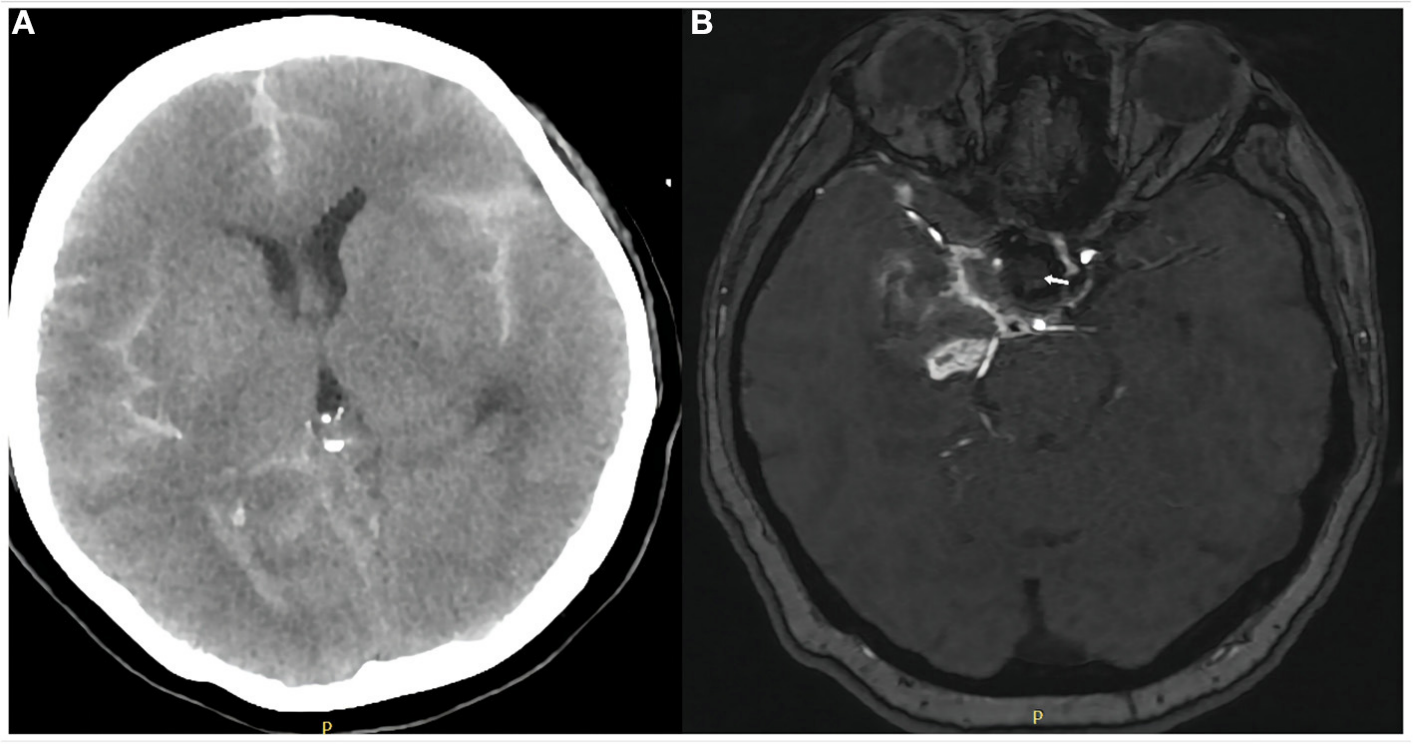
Computed tomography of the head on day 1 after covered stent placement **(A)** showed no progression of subarachnoid hemorrhage or other obvious abnormality. Magnetic resonance angiography of the brain on day 6 **(B)** showed the aneurysm was completely occluded (white arrow) and the parent artery was patent.

The patient was extubated on the subsequent day after repeat head CT showed no progression of SAH (Figure 4A). Patients were asked to continue dual antiplatelet therapy (clopidogrel 75 mg/day and aspirin 100 mg/day) for at least 6 months after it was determined that the bleeding had stopped. Aspirin monotherapy was continued for life. Magnetic resonance angiography 6 days after treatment demonstrated parent artery patency and no aneurysmal filling (Figure 4B). The patient's left limb strength was grade 3 on manual muscle testing. She was transferred to a rehabilitation facility 1 week after WCS placement with modified Rankin scale score 2. However, we followed up with her by phone after 1 month and there were no new symptoms and the mRS score was 1, which was an improvement from the time of discharge.

## Discussion

Flow diversion is widely used to treat large and giant intracranial aneurysms. However, delayed aneurysmal rupture (DAR) is a potentially devastating complication (8). In previous studies, reported incidence rates of DAR after FD placement ranged between 0.6 and 4% (9). The multicenter Chinese PLUS study reported an incidence of 2.1% (3). Approximately 80% of DARs occur within 30 days of the procedure. DAR results in poor clinical outcome or death in 70 to 80% of cases (9). Although the mechanism underlying DAR is unknown, computational fluid dynamic studies have demonstrated that flow modifications after stent placement can result in intra-aneurysmal pressure increases that may lead to rupture, especially in large and giant aneurysms (10). Other studies have suggested a potential role of intra-aneurysmal thrombus, as the thrombus is a source of various proteases with high proteolytic activity that can degrade the arterial wall (11, 12). Hou et al. (13) reported that 88.8% of the aneurysms that experienced delayed rupture after FD placement were large or giant; moreover, 97.8% were symptomatic. Saccular aneurysms with aspect ratio >1.6 also have a higher risk of DAR (11). Intra-thrombus proteases may play a role in the delayed rupture of these aneurysms, as they are more likely to have intraluminal thrombus (4, 9, 13–15). In large or giant aneurysms, adjunctive coiling or placement of multiple FDs to achieve complete aneurysm thrombosis and early isolation from the circulation have been suggested because of the higher risk of delayed rupture (16, 17). However, these strategies are not a panacea: in one study of patients presenting with DAR, multiple FDs were placed in 38.3% of patients (13); in another, adjunctive coiling was used in 20% (9, 13).

DAR can present with acute intracranial bleeding or carotid–cavernous sinus fistula (CCF). Rupture of ICA aneurysms located above the ophthalmic segment (C6) cause intracranial hemorrhage, while rupture of those located below the clinoid segment (C5) usually cause CCF. Patients presenting with CCF after FD placement tend to experience a relatively more favorable outcome; in contrast, those who present with hemorrhage almost always die because they deteriorate rapidly before further treatment can be rendered (13). Even with aggressive management, most patients experience inevitable deterioration and death. To the best of our knowledge, a standard treatment of DAR after FD placement has not been established. However, all patients are at risk because the treatment effect of flow diversion is not immediate but occurs over time. Mazur et al. (18) reported attempted implantation of a second FD in a patient who experienced DAR after placement of a single FD, but unfortunately failed. Hence, if rupture occurs during the latency period, the aneurysm must be isolated from the circulation as soon as possible to avoid rehemorrhage.

The WCS is a parent vessel reconstruction tool that can exclude an aneurysm from the circulation and achieve immediate occlusion. In theory, the WCS is the best treatment for intracranial aneurysms by preventing blood flow into the aneurysm sac and preserving the parent artery, thereby restoring normal vascular morphology. The use of WCS also avoids direct manipulation in the aneurysm sac, reducing the risk of aneurysm rupture (19). Willis covered stents have been proven to be safe and effective in the treatment of ruptured or unruptured ICA siphon aneurysms, distal ICA aneurysms, recurrent intracranial aneurysms after coiling, and large or giant aneurysms (5, 20–22). However, Zhu et al. (23) also noted that it is not recommended for patients with a tortuous parent artery or critical side branches, particularly the anterior choroidal artery, associated with the aneurysm. In view of this, PFED treatment was chosen instead of WCS treatment in our patient first. However, in our patients with DAR after PFED placement resulting in subarachnoid hemorrhage, we used WCS in addition to the original PFED in order to isolate the ruptured aneurysm as soon as possible. This occluded the aneurysm, prevented further hemorrhage, and resulted in a favorable clinical outcome. Covered stent placement is a feasible treatment option for patients with DAR after FD placement. However, its long-term outcome remains to be further followed up.

## Author's note

This work originated from the Beijing Neurosurgical Institute and Beijing Tian Tan Hospital, South Fourth Ring Road West 119, Fengtai District, Beijing.

## Data availability statement

The datasets presented in this article are not readily available because of ethical and privacy restrictions. Requests to access the datasets should be directed to the corresponding author/s.

## Ethics statement

The studies involving human participants were reviewed and approved by the Ethics Committee of Beijing Tian Tan Hospital. Written informed consent to participate in this study was provided by the patients/participants. Written informed consent was obtained from the individual(s) for the publication of any potentially identifiable images or data included in this article.

## Author contributions

XC and SG wrote the first draft of the manuscript and acquired the data. LZ and LD analyzed and interpreted the data. HG and PL edited the figures of the article. YL and ML conceived and designed the research. All authors contributed to the article and approved the submitted version.

## Funding

This study was supported by the National Natural Science Foundation of China (Grant No. 82171289) and Youth Program of National Natural Science Foundation of China (Grant No. 81901197).

## Conflict of interest

The authors declare that the research was conducted in the absence of any commercial or financial relationships that could be construed as a potential conflict of interest.

## Publisher's note

All claims expressed in this article are solely those of the authors and do not necessarily represent those of their affiliated organizations, or those of the publisher, the editors and the reviewers. Any product that may be evaluated in this article, or claim that may be made by its manufacturer, is not guaranteed or endorsed by the publisher.
